# Hypotrichosis and juvenile macular dystrophy caused by *CDH3* mutation: A candidate disease for retinal gene therapy

**DOI:** 10.1038/srep23674

**Published:** 2016-05-09

**Authors:** Mandeep S. Singh, Suzanne Broadgate, Ranjana Mathur, Richard Holt, Stephanie Halford, Robert E. MacLaren

**Affiliations:** 1 Nuffield Laboratory of Ophthalmology, Nuffield Department of Clinical Neuroscience, University of Oxford, Oxford, United Kingdom; 2 Singapore National Eye Centre and Singapore Eye Research Institute, Singapore; 3 Oxford Eye Hospital, Oxford University Hospitals NHS Trust and NIHR Biomedical Research Centre, Oxford, UK; 4 Moorfields Eye Hospital NHS Foundation Trust and NIHR Ophthalmology Biomedical Research Centre, London, UK

## Abstract

Hypotrichosis with juvenile macular dystrophy (HJMD) is an autosomal recessive disorder that causes childhood visual impairment. HJMD is caused by mutations in *CDH3* which encodes cadherin-3, a protein expressed in retinal pigment epithelium (RPE) cells that may have a key role in intercellular adhesion. We present a case of HJMD and analyse its phenotypic and molecular characteristics to assess the potential for retinal gene therapy as a means of preventing severe visual loss in this condition. Longitudinal *in vivo* imaging of the retina showed the relative anatomical preservation of the macula, which suggested the presence of a therapeutic window for gene augmentation therapy to preserve visual acuity. The coding sequence of *CDH3* fits within the packaging limit of recombinant adeno-associated virus vectors that have been shown to be safe in clinical trials and can efficiently target RPE cells. This report expands the number of reported cases of HJMD and highlights the phenotypic characteristics to consider when selecting candidates for retinal gene therapy.

Hypotrichosis with juvenile macular dystrophy (HJMD, OMIM 601553) is a rare congenital autosomal recessive disorder causing visual impairment that begins in childhood^1^. HJMD is caused by loss-of-function mutations in *CDH3* (NM_001793)^2^ which encodes cadherin-3 (also called P-cadherin), a critical mediator of cell-cell adhesion^3^ which is expressed in human retinal pigment epithelium (RPE)^4^. In the eye, abnormal or absent *CDH3* expression is postulated to affect RPE monolayer arrangement^5^, causing progressive macular degeneration and visual loss. In the current era of retinal gene therapy, here we analyse the longitudinal clinical characteristics in HJMD to ascertain if a therapeutic window exists for the application of retinal gene therapy in the future.

## Results

**Figure 1:**
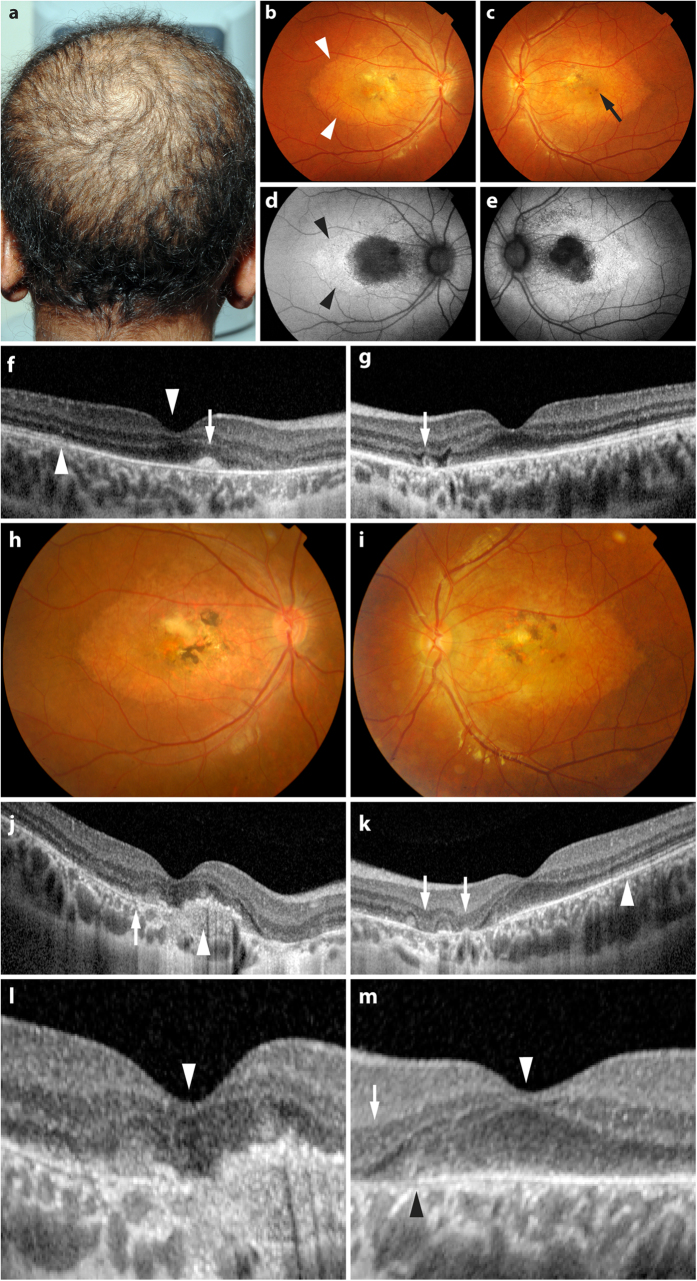
(**a**) The patient had sparse and short hair. (**b**,**c**) Hypopigmented areas (arrowheads in **b**) of retinal pigment epithelial (RPE) cells and small foveal pigment clumps (arrow in **c**) were present bilaterally. (**d**,**e**) Foveal autofluorescence was reduced, reflecting loss of RPE cells. (**f**) Spectral domain optical coherence tomography (SDOCT) imaging showed preserved right foveal center thickness (downward arrowhead). A small hyperintense deposit corresponding to a pigment clump occupies the subretinal space approximately 440 μm nasal to the foveal center (arrow). (**g**) SDOCT in the left fovea showing preserved foveal center thickness. A small outer retinal tubulation approximately 1050 μm nasal to the fovea (arrow) indicated localised photoreceptor loss. (**h**,**i**) Three years later, there were more advanced degenerative changes. (**j**) SDOCT of the right eye three years post baseline showing focal photoreceptor loss (arrow) and subretinal pigment epithelium scar (arrowhead), but relative preservation of the central foveal thickness (magnified in **l**, arrowhead). (**k**) SDOCT of the left eye three years post baseline, with focal ONL loss (arrows) outside the foveal center. (**m**) Magnified view of the right foveal center from (**k**) showing preserved central foveal thickness (arrowhead) and the presence of the subfoveal RPE temporal to the black arrowhead. The arrow denotes focal photoreceptor loss outside the foveal center. The inner segment/outer segment (IS/OS) line at the foveal center is absent in both (**l**,**m**).

The patient was an 11-year-old boy with blurred vision in his right eye for one year. He had sparse hair on his scalp (Fig. 1a). There were no other affected family members. His visual acuity was 20/30 in the right eye and 20/25 in the left. Clinical examination revealed features of macular dystrophy including symmetrical areas of RPE hypopigmentation extending from the optic discs to the temporal maculae in the right and left fundi (Fig. 1b,c). Retinal flecks were absent. There were small hyperpigmented RPE clumps centered on each fovea. Autofluorescence (AF) was increased in the zone of RPE hypopigmentation detected clinically but reduced at the foveal centers, reflecting central RPE cell loss (Fig. 1d,e).

In the right macula at baseline (Fig. 1f), spectral domain optical coherence (SDOCT) imaging showed outer retinal abnormalities including a hyperintense subretinal deposit and focal outer nuclear layer (ONL) thinning outside the foveal center, representing focal loss of photoreceptor cells. Despite the abnormal anatomy of subfoveal RPE interdigitation zone (IZ) and the inner segment/outer segment (IS/OS) junction SDOCT lines, the foveal center thickness appeared relatively normal. In the left eye (Fig. 1g), baseline SDOCT imaging revealed normal foveal thickness, albeit with loss of the normal subfoveal IZ and IS/OS lines, and a focus of subretinal tubulation indicating localised photoreceptor degeneration. He was clinically diagnosed with juvenile macular dystrophy associated with alopecia, with foveal sparing.

Three years later, the patient’s visual acuity had declined to 20/80 and 20/40 in the right and left eyes respectively. There was clinical evidence of more advanced macular atrophy, subretinal scarring, and pigment clumping involving the juxtafoveal zones in both eyes (Fig. 1h,i). Fundus fluorescein angiography excluded choroidal neovascularisation (data not shown) as a cause of the visual decline. SDOCT imaging (Fig. 1j–m) showed more advanced degenerative retinal changes, however central foveal thickness remained within the normal range despite the degeneration of subfoveal RPE cells.

**Figure 2:**
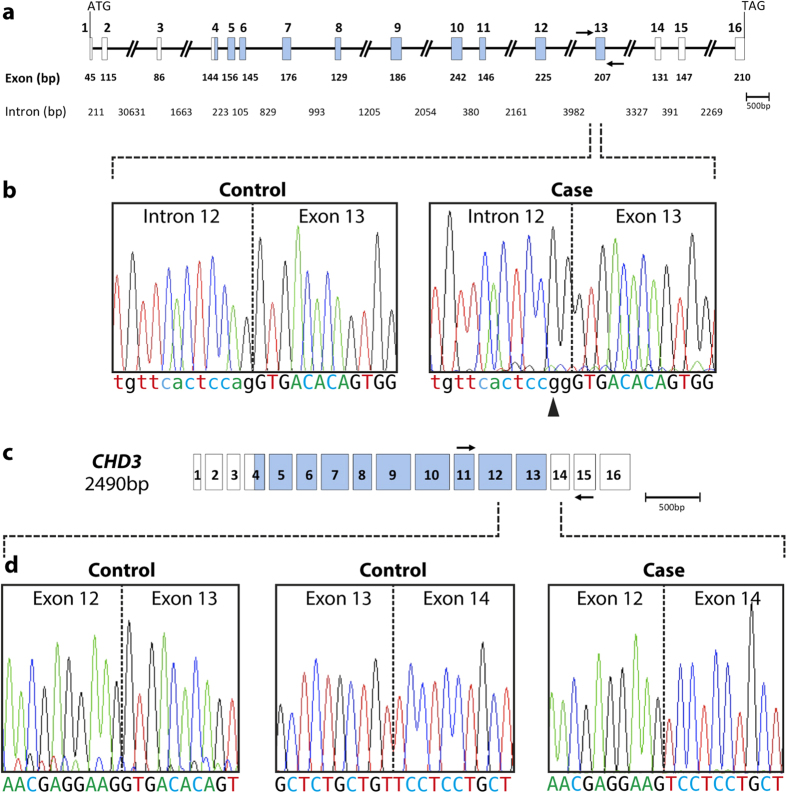
(**a**) Schematic diagram of the genomic structure of *CDH3*. Exons are depicted as boxes and introns as lines; all are to scale except for regions of intronic DNA larger than 1kb that are represented as slashed lines. Exon and intron sizes are marked, as are the start (ATG) and stop (TAG) codons. The exons encoding the calcium binding domains are shaded in blue. Arrows mark the approximate position of the intronic primers used for PCR of exon 13. (**b**) Sequence of the intron 12/exon13 boundary in a control sample and the patient, a homozygous change in intron 12 (c.1796-2A>G) is seen in the case (arrowhead). Dotted lines mark the intron-exon boundary. Intronic sequences are shown in lower case lettering. (**c**) Schematic diagram of the 2490bp *CDH3* mRNA. The arrows mark the approximate position of the exonic primers used for PCR in exon 11 and exon 15. (**d**) The sequence obtained from the PCR fragment obtained from cDNA, providing confirmation that the base change shown in (**a**) results in a product that was missing exon 13 in the patient. The exon 12/exon 13 and exon 13/exon 14 boundaries in a control is shown alongside the sequence obtained in the patient, where exon 12 is spliced directly to exon 14. Dotted lines mark the exon boundaries.

All 16 exons of the *CDH3* gene were amplified and sequenced. A recurrent homozygous mutation^6^ in intron 12 (c.1796-2A>G) was detected in the patient (Fig. 2a,b). This mutation in the splice acceptor site is predicted to cause aberrant splicing and the loss of exon 13 in the mRNA product. RNA was extracted from peripheral blood lymphocytes and cDNA was synthesised to test the predicted effect of this mutation on splicing. Primers were designed to span several exons, and a product was generated from exon 11 to exon 15 in the patient and a control. The predicted fragment of 611 bp was generated from the control, however a smaller fragment (404 bp) was observed in the patient. Sanger sequencing confirmed the loss of exon 13 in the patient sample (Fig. 2c,d). The homozygous mutation found in this patient, causing skipping of exon 13, is predicted to result in a truncated *CDH3* protein causing the loss of the extracellular calcium binding domain and part of the transmembrane domain.

## Discussion

Gene therapy for retinal degenerations has been evaluated in several clinical trials^7^,^8^,^9^,^10^, however the phenotypic characteristics that should guide patient selection for macular gene therapy are not fully defined. Among the prerequisites for a macular dystrophy to be considered a target for gene therapy is a natural history that permits a time window for treatment. In the patient described here, visual acuity had declined gradually over the three years of follow up. In this interval, the anatomy of the left fovea is striking for the absence of scar and the relative preservation of central foveal thickness despite the presence of focal areas of progressive photoreceptor degeneration outside the foveal center. This suggests the presence of macular photoreceptor cells that could, in principle, be rescued from further degeneration by the administration of *CDH3* gene augmentation therapy to the remaining RPE cells in the macula. SDOCT findings in a 6-year-old boy with reduced visual acuity due to HJMD^11^ demonstrated preserved central macular thickness in the absence of subretinal and sub-RPE scars. Taken together with the longitudinal imaging data presented here, we infer that gene augmentation therapy, if applied at the stage when foveal photoreceptors and RPE cells survive as targets, may be successful in preventing or retarding progressive visual loss in HJMD. A report of 16 HJMD cases^12^ suggested a progressive decline in some cases over time. Moreover, some young patients can show normal ERG responses^11^. Hence, the generally slow progression of decline in macular dystrophy due to HJMD also implies a time window for retinal gene therapy.

Retinal gene therapy clinical trials thus far reported have employed recombinant AAV (rAAV) vectors to deliver therapeutic DNA into target retinal cells. A limiting factor in the list of candidate diseases that could be treated with rAAV gene therapy is the size of the cDNA sequence of the mutated gene. The cargo of rAAV vectors is limited to approximately 4.7 kB of single stranded DNA including promoter and inverted terminal repeat sequences. The coding sequence of *CDH3* spans approximately 2.5 kb, and can therefore be delivered using rAAV vectors. Significantly, rAAV type 2 (rAAV2) has been shown to have a favorable safety profile in retinal gene therapy in clinical trials^7^,^8^,^9^,^10^. Gene therapy with rAAV2, using a RPE-specific promoter, has been shown to produce efficient and exclusive gene expression in RPE cells, and has been evaluated for use in a gene therapy trial for retinitis pigmentosa due to mutations in *MERTK* (NM_006343), an RPE-expressed gene^13^. As HJMD is caused by the loss of function of cadherin-3 in RPE cells, it is amenable to a curative approach of restoring the expression of normal *CDH3* by targeting rAAV2 vectors bearing the therapeutic DNA to RPE cells in the retina.

Although macular dystrophy is a consistent feature, phenotypic variability is known to occur in HJMD^14^. Hence, candidacy for gene therapy is likely to be dependent on specific features (such as the extent of foveal center sparing) in each case. Few other reports of this condition exist in the literature^2^,^6^,^12^,^15^,^16^,^17^,^18^,^19^. This report expands the number of reported cases of HJMD, suggests the condition as a candidate disease for gene therapy, and highlights the phenotypic characteristics to consider when selecting patients for gene therapy of the macula in the future.

## Methods

This research was approved by the local ethics committee and was conducted in accordance with the principles of the Declaration of Helsinki. Informed consent was obtained. Genomic DNA was extracted from peripheral blood (QIAamp DNA blood midi kit, Qiagen UK). Each of the 16 exons of *CDH3* was amplified from the genomic DNA using primers located in flanking introns and untranslated regions. Polymerase chain reaction (PCR) was performed using a published protocol^20^, amplifying exons and splice junctions. RNA was extracted (QIAamp RNA blood mini kit, Qiagen, UK) and cDNA synthesised from 300 ng RNA (Quantitect kit, Qiagen, UK). A fragment spanning exon 11 to exon 15 was amplified as follows: 94 °C for 5 minutes, 94 °C for 30 s, 62 °C for 30 s, 72 °C for 1 min for 35 cycles, and 72 °C for 10 minutes. Each 25 μl reaction contained 12.5 μl Biomix (Bioline Ltd), 0.2 μM^6^ of each primer, 1 μl glycerol and 1 μl cDNA from the patient and a control as template. The primers *CDH3*ex11F (5′-GTGAGGATGAGCAGTTTGTG-3′) and *CDH3*ex15R (5′-TGTCGGGATGATGGTTGGTG-3′) were expected to generate a 716 bp fragment. A second round of PCR was performed using nested primers, *CDH3*ex11F2 (5′-GAAGTCATGGTCTTGGCCAT-3′) and *CDH3*ex15R2 (5′-AGCTGGGTGATGTCATAGTC-3′) to generate a 611 bp fragment. Conditions were: 94 °C for 5 minutes, 94 °C for 30 s, 56 °C for 30s, 72 °C for 45 s for 35 cycles, 72 °C for 10 minutes. Each 25 μl reaction contained 12.5 μl Biomix (Bioline Ltd), 0.2 μM of each primer, and 1 μl 1:10 diluted first round product from the patient and a control as template. The products were Sanger sequenced using forward and reverse primers.

## Additional Information

**How to cite this article**: Singh, M. S. *et al*. Hypotrichosis and juvenile macular dystrophy caused by *CDH3* mutation: A candidate disease for retinal gene therapy. *Sci. Rep*. **6**, 23674; doi: 10.1038/srep23674 (2016).

## References

[b1] Souied E. . Unusual association of juvenile macular dystrophy with congenital hypotrichosis: occurrence in two siblings suggesting autosomal recessive inheritance. Ophthalmic Genet. 16, 11–15, doi: 10.3109/13816819509057848 (1995). 7648037

[b2] Sprecher E. . Hypotrichosis with juvenile macular dystrophy is caused by a mutation in *CDH3*, encoding P-cadherin. Nat. Genet. 29, 134–136, doi: 10.1038/ng716 (2001). 11544476

[b3] Shimomura Y., Wajid M., Shapiro L. & Christiano A. M. P-cadherin is a p63 target gene with a crucial role in the developing human limb bud and hair follicle. Development 135, 743–753, doi: 10.1242/dev.006718 (2008). 18199584

[b4] Strunnikova N. V. . Transcriptome analysis and molecular signature of human retinal pigment epithelium. Hum. Mol. Genet. 19, 2468–2486, doi: 10.1093/hmg/ddq129 (2010). 20360305 PMC2876890

[b5] Burke J. M., Cao F., Irving P. E. & Skumatz C. M. Expression of E-cadherin by human retinal pigment epithelium: delayed expression *in vitro*. Invest. Ophthalmol. Vis. Sci. 40, 2963–2970 (1999). 10549658

[b6] Shimomura Y., Wajid M., Kurban M. & Christiano A. M. Splice site mutations in the P-cadherin gene underlie hypotrichosis with juvenile macular dystrophy. Dermatology 220, 208–212, doi: 10.1159/000275673 (2010). 20203473 PMC2865484

[b7] Cideciyan A. V. . Human gene therapy for RPE65 isomerase deficiency activates the retinoid cycle of vision but with slow rod kinetics. Proc. Natl. Acad. Sci. USA 105, 15112–15117, doi: 10.1073/pnas.0807027105 (2008). 18809924 PMC2567501

[b8] Bainbridge J. W. B. . Effect of gene therapy on visual function in Leber’s congenital amaurosis. N. Engl. J. Med. 358, 2231–2239, doi: 10.1056/NEJMoa0802268 (2008). 18441371

[b9] Maguire A. M. . Safety and efficacy of gene transfer for Leber’s congenital amaurosis. N. Engl. J. Med. 358, 2240–2248, doi: 10.1056/NEJMoa0802315 (2008). 18441370 PMC2829748

[b10] MacLaren R. E. . Retinal gene therapy in patients with choroideremia: initial findings from a phase 1/2 clinical trial. Lancet 383, 1129–1137, doi: 10.1016/s0140-6736(13)62117-0 (2014). 24439297 PMC4171740

[b11] Mason J. O. & Patel S. A. A case of hypotrichosis with juvenile macular dystrophy. Retin Cases Brief Rep, doi: 10.1097/icb.0000000000000127 (2015). 25621871

[b12] Leibu R. . Hypotrichosis with juvenile macular dystrophy: clinical and electrophysiological assessment of visual function. Ophthalmology 113, 841–847. e843, doi: 10.1016/j.ophtha.2005.10.065 (2006). 16650681

[b13] Conlon T. J. . Preclinical potency and safety studies of an AAV2-mediated gene therapy vector for the treatment of MERTK associated retinitis pigmentosa. Human Gene Therapy Clinical Development 24, 23–28, doi: 10.1089/humc.2013.037 (2013). 23692380 PMC3856558

[b14] Indelman M. . Phenotypic diversity and mutation spectrum in hypotrichosis with juvenile macular dystrophy. The Journal of Investigative Dermatology 121, 1217–1220, doi: papers2://publication/doi/10.1046/j.1523-1747.2003.12550_1.x (2003). 14708629 10.1046/j.1523-1747.2003.12550_1.x

[b15] Halford S., Holt R., Nemeth A. H. & Downes S. M. Homozygous deletion in *CDH3* and hypotrichosis with juvenile macular dystrophy. Arch. Ophthalmol. 130, 1490–1492, doi: 10.1001/archophthalmol.2012.708 (2012). 23143461

[b16] Indelman M. . Novel *CDH3* mutations in hypotrichosis with juvenile macular dystrophy. Clin. Exp. Dermatol. 32, 191–196, doi: 10.1111/j.1365-2230.2006.02335.x (2007). 17342797

[b17] Indelman M., Leibu R., Jammal A., Bergman R. & Sprecher E. Molecular basis of hypotrichosis with juvenile macular dystrophy in two siblings. Br. J. Dermatol. 153, 635–638, doi: 10.1111/j.1365-2133.2005.06734.x (2005). 16120155

[b18] Jelani M., Salman Chishti M. & Ahmad W. A novel splice-site mutation in the *CDH3* gene in hypotrichosis with juvenile macular dystrophy. Clin. Exp. Dermatol. 34, 68–73, doi: 10.1111/j.1365-2230.2008.02933.x (2009). 19076794

[b19] Kamran-ul-Hassan Naqvi S., Azeem Z., Ali G. & Ahmad W. A novel splice-acceptor site mutation in *CDH3* gene in a consanguineous family exhibiting hypotrichosis with juvenile macular dystrophy. Arch. Dermatol. Res. 302, 701–703, doi: 10.1007/s00403-010-1035-6 (2010). 20140736

[b20] Kjaer K. W. . Distinct *CDH3* mutations cause ectodermal dysplasia, ectrodactyly, macular dystrophy (EEM syndrome). J. Med. Genet. 42, 292–298, doi: 10.1136/jmg.2004.027821 (2005). 15805154 PMC1736041

